# Epidemiology and disease burden of androgenetic alopecia in college freshmen in China: A population-based study

**DOI:** 10.1371/journal.pone.0263912

**Published:** 2022-02-16

**Authors:** Fanping He, Minxue Shen, Zhixiang Zhao, Yicong Liu, Shuping Zhang, Yan Tang, Hongfu Xie, Xiang Chen, Ji Li

**Affiliations:** 1 Department of Dermatology, Xiangya Hospital of Central South University, Changsha, Hunan, China; 2 Hunan Key Laboratory of Aging Biology, Xiangya Hospital, Central South University, Changsha, China; 3 National Clinical Research Center for Geriatric Disorders, Xiangya Hospital of Central South University, Changsha, Hunan, China; Chiba Daigaku, JAPAN

## Abstract

**Objective:**

To evaluate the epidemiology and disease burden of androgenetic alopecia (AGA) in college freshmen in China.

**Methods:**

This population-based cross-sectional survey was carried out among 9227 freshmen of two comprehensive universities in two cities of China (Changsha and Xiamen) from September 2018 to October 2018. Questionnaires covering basic issues, surrounding demographic information, history of diseases, living habits, comorbidities, etc. were completed online in a self-reported manner Dermatological examination was performed by certified dermatologists. The disease burden of AGA, which includes health-related quality of life, symptoms of anxiety, symptoms of depression and quality of sleep, was measured by EQ-5D-3L, PHQ-2, GAD-2 and PSQI, respectively.

**Results:**

The prevalence of AGA in college freshmen in China was 5.3/1000. Male was significantly associated with higher prevalence of AGA (7.9/1000, P<0.01) while female with lower risk of AGA (OR = 0.29, P = 0.002). There was no significant association between BMI and AGA, nor predilection of AGA in the Han nationality or the other ethnic minorities. Annual household income or parental highest educational level exerted no significant influence on the prevalence of AGA. Rosacea (OR = 3.22, P = 0.019) was significantly associated with higher prevalence of AGA while acne seemed not to be related to AGA. The scores of EQ-5D, GAD-2, PHQ-2 and PSQI were not significantly different between students with and without AGA.

**Conclusion:**

The onset of AGA in Chinese college freshmen differ between genders and was significantly associated with rosacea.

## 1. Introduction

Androgenetic alopecia (AGA) is the most common type of hair loss in the world and is usually caused by multiple factors. Typically, AGA is distributed in genetically susceptible men and women in specific patterns-male pattern and female pattern hair loss (MPHL and FPHL). The characteristic finding of MPHL is frontoparietal and frontal hairline recession, followed by a vertex thinning with progression until the top of the scalp is completely bald [1]. FPHL generally manifests as a diffuse thinning of the centroparietal region with preservation of the frontal hairline [2]. In men, the onset of AGA is specifically related to androgen hyperactivity and genetic factors, while female AGA may have a more complex etiology which is not well understood [3, 4].

Epidemiology of AGA has been extensively studied. In general, AGA is affecting about 0.2–2% of all the populations in the world and the prevalence of AGA is known to differ between genders and races [5–7]. What’s more, the incidence rate and severity of AGA were increasing with age in all genders and races [3, 5, 8, 9]. A epidemiological surveys in China reported that the prevalence of AGA increased from 2.8% and 1.3% in men and women aged 18–29 to 41.4% and 11.8% aged older than 70 [5]. However, no previous study has been performed focusing on the epidemiology of AGA in the college students.

Previous studies implied that the prevalence of AGA varied between cities in China which might be due to the difference in climate, life-style, or economic levels [5]. However, the exact relationship between AGA and lifestyle, living habits or economic level has not been studied before. Beside, several studies revealed that androgenetic alopecia may be associated with hypertension, metabolic syndrome, central obesity and other cardiovascular risk factors [10]. For specific, the work by Ozturk et al. proved that body mass index (BMI) of AGA patients were higher than control group [11]. Consistently, Fortes et al. also showed that the combination of overweight and smoking is associated with an increased severity of androgenetic alopecia [12]. However, there are also other studies demonstrating that there was no significant association between these cardiovascular risk factors (such as hypertension, smoking, BMI) and AGA [10, 13]. For college students, it is quite rare for them to have hypertension or other cardiovascular diseases at their age. However, they are prone to be implicated with skin diseases such as acne vulgaris and rosacea. The possible relationship between AGA and acne or rosacea were suggested since anti-androgenic therapy has been shown to be effective not only for AGA, but also for acne and rosacea [14, 15]. Therefore, rosacea or acne may be associated with AGA due to an underlying state of hyperandrogenism [14, 16, 17]. As a result, it would be interesting to explore the possible risk factors and comorbidities (especially skin diseases such as acne vulgaris and rosacea) of AGA in college students.

The disease burden of AGA is usually measured by several different dimensions including health-related quality of life, anxiety, depression, and quality of sleep, etc. Previous studies suggested that hair loss can have a significant effect on health-related quality of life (HrQoL) [18–21]. For many people, thick hair might be positively relevant to social status, femininity power and self-confidence. A recent comprehensive review study among alopecia patients concluded that the quality of life of patients were significantly impaired by AGA, which was more severe in female [21]. Similarly, another research of 315 subjects in a wide age range (18–81 years) found a high prevalence of anxiety, depression and other minor psychiatric in AGA patients, with a significantly higher frequency in female (60%) [22]. What’s more, the study by Yamazaki M et al. suggested that oral finasteride do have improved the QoL of AGA patients despite no relieve of their anxieties [23]. Hence, it might be helpful to provide additional psychological counseling by dermatologists while treating AGA patients, especially in the early stage of the disease. However, to date there is rarely researches concerning the comprehensive disease burden of AGA in adolescents.

## 2. Methods

### 2.1 Study design

This is a population-based cross-sectional survey. Participants were newly enrolled college students (13,612) who consented to participate in two comprehensive universities in two cities of China (Changsha 8676 and Xiamen 4936), with geographically dispersed enrollment policy. Sixty-eight percent of them (9,227 of 13612) consented to participate, finished the online questionnaire surveys in a self-reported manner, and accepted the dermatological examination on common skin diseases by certified dermatologists through September and October 2018.

The departments of student affairs of the universities organized the questionnaire survey. The questionnaire consisted of several questions that covered basic issues, surrounding demographic information, history of diseases, living habits, etc. and was fulfilled through a web-based survey system. Research nurses using standardized methods measured height and weight. Body mass index (BMI) was calculated as weight (kg) divided by the square of height (m^2^). More details can be found in previous publications of the pilot study in 2017 [24, 25].

### 2.2 Clinical evaluations and diagnosis

Generally, definitive diagnosis of AGA is based on patient history and a thorough scalp examination. The diagnosis and associated disease history were performed by qualified dermatologists with extensive clinical experience during the dermatological examinations. All the AGA patients were graded according to Hamilton-Norwood classification.

Health-related quality of life (HrQoL) was assessed via validated Chinese version of EQ-5D-3L. The EQ-5D-3L assesses health status through three response levels of mobility, self-care, usual activities, pain/discomfort and anxiety/depression. Further information is available at www.euroqol.org. Symptoms of anxiety and depression were respectively evaluated by the two-item Generalized Anxiety Disorder Scale (GAD-2) and two-item Patient Health Questionnaire (PHQ-2) with good sensitivity and specificity [26]. GAD-2 and PHQ-2 are ultra-brief and valid scales consist two questions reflecting DSM-V core diagnostic criteria, are the abridged version of GAD-7 and PHQ-9. The stem items of GAD-2 based on feeling nervous and controlling worrying. The stem items of PHQ-2 are depressing mood and anhedonia. Quality of sleep was evaluated by the Pittsburgh Sleep Quality Index (PSQI) which components seven score: subjective sleep quality, sleep latency, sleep duration, habitual sleep efficiency, sleep disturbances, use of sleeping medication, and daytime dysfunction [27].

All experimental protocols were approved by Ethics Committee of Xiangya Hospital of Central South University. All methods were carried out in accordance with relevant guidelines and regulations. Informed consent was obtained from all subjects in the online questionnaire.

### 2.3 Statistical analysis

The method was validated in Chinese patients. Continuous data are presented as the mean ± standard deviation, and between-group differences were tested using analysis of variance (ANOVA). Categorical data are presented as number (%), and between-group differences were tested using the chi-square test. The associations of demographic factors (age, gender, ethnicity, annual household income, parental highest educational level) and BMI with AGA were analyzed using mixed logistic models (student as level-1 unit and province as level-2 unit).

The effect of AGA on quality of life was analyzed. The utility estimate was mapped from EQ-5D-3L using the method proposed by Liu GG *et al*. [28]. EQ-5D-3L responses into not impaired (‘no problems’) and impaired (‘some problems’ or ‘extreme problems’). The scores of GAD-2 and PHQ-2 options were computed by adding scores of the response: not at all (score = 0), several days (score = 1), more than half the days (score = 2), and nearly every day(score = 3). The recommended cut points is ≥3. The higher the score, the more likely suffering depressive or anxiety disorder. PSQI score was computed as the sum of seven components and was dichotomized by cutoff ≥6 according to a previous validation study in U.S. college students. Odds ratios (ORs) and 95% confidence intervals (CIs) were estimated from the model. Statistical analysis was performed in SAS 9.4 (SAS Inc., Cary, USA). *P*<0.05 was considered statistically significant.

## 3. Results

### 3.1 Prevalence and characteristics of AGA in college freshmen in China

A total of 9,227 participants consented to participate, completed the online questionnaire survey and underwent the dermatological examination. The mean age of the participants was 18.2±0.7 years old, and 52% of them were male.

The characteristics of the participants are shown in Table 1. The results revealed that the prevalence of AGA was 5.3/1000. It is apparent from this table that male was significantly associated with higher prevalence of AGA (*P*<0.01), which was consistent with the previous study [8].

**Table 1:** Prevalence of androgenetic alopecia by characteristics of participants.

Variables		Androgenetic alopecia			
	Total	Present	Clear	*P*	*P*_*trend*_
N(%)	9227	49(0.53)	9178(99.47)		
**Age (years±SD)**	18.2±0.7	18.29±0.71	18.19±0.69	0.328	
≤16 years	44	1	43		
17 years	806	3	803		
18 years	6092	27	6065		
19 years	1994	17	1977		
20 years	244	1	243		
≥ 21 years	47	0	47		
Body mass index (kg/m^2^)	21.1±3.4	21.91±3.91	21.09±3.36	0.087	
**Gender**					
Male	4799	38 (0.79)	4761 (99.2)	***<0*.*001***	
Female	4428	11 (0.25)	4417 (99.75)		
**Ethnicity**					
Han	8240	42 (0.51)	8198 (99.49)	0.415	
Other	987	7 (0.71)	980 (99.29)		
**Annual household income (CNY)**					
< 30,000	2294	15 (0.65)	2279 (99.35)	0.646	0.424
30,000–99,999	3641	18 (0.49)	3623 (99.51)		
≥ 100,000	3292	16 (0.49)	3276 (99.51)		
**Parental highest educational level**					
Middle school and below	2685	16 (0.60)	2669 (99.40)	0.853	0.587
High school	2309	12 (0.52)	2297 (99.48)		
College and above	4233	21 (0.50)	4212 (99.50)		
**Classification (Hamilton-Norwood)**					
I		38 (0.78)			
II		1 (0.02)			
III		7 (0.14)			
IV		1 (0.02)			
V		2 (0.04)			
**Acne vulgaris**					
Clear / mild	8140	39 (0.48)	8101 (99.52)	0.060	
Moderate / Severe	1087	10 (0.92)	1077 (99.08)		
**Rosacea**					
Clear	8917	44 (0.49)	8873 (99.51)	0.003	***<0*.*001***
Rosacea	310	5 (1.61)	305 (98.39)		

BMI: Body mass index.

In contrast to some previous studies, the findings of our study did not show a significant association between BMI and AGA. There was no predilection of AGA in the Han nationality or the ethnic minorities, as shown in Table 1. Annual household income, parental highest educational level, living habits or lifestyle exerted no influence on the prevalence of AGA.

Since the allergic disease, endocrine disorder, hematological disease, infectious disease, immunological disease, cardiovascular disease or its risk factors especially smoking we studied in the questionnaire were quite rare among freshmen in college, the number was too limited to draw any conclusion. We also performed thorough dermatological examination on the common skin diseases which reveal that the most common comorbidities of AGA were acne vulgaris and rosacea. We explored the relationship between several state of acne vulgaris (Clear/ mild and Moderate/ Severe) or rosacea (clear/rosacea) with AGA. The results suggested that rosacea was significantly related to AGA (*p* for trend<0.001). There is no significant relationship between the onset of AGA and acne vulgaris.

### 3.2 Risk factors and comorbidities of AGA in college freshmen in China

The associations between AGA with the characteristics of participant were estimated using a mixed logistic model (Table 2). Consistent with crude estimates, female was associated with lower risk of AGA (OR = 0.29, *P* = 0.002). Clear of rosacea was set as the reference group, compared with which, rosacea (OR = 3.22, *P* = 0.019) were significantly associated with higher prevalence of AGA. Similar to the analysis shown in Table 1, there was no direct correlation between AGA with ethnicity, BMI, annual household income, parental highest education level or acne vulgaris.

**Table 2:** Association of androgenetic alopecia with characteristics of participants.

Variables	AOR (95% CI)	*P*
Age (years)	1.11 (0.76–1.61)	0.605
Body mass index (kg/m^2^)	1.03 (0.96–1.12)	0.387
**Gender**		
Male	Ref	
Female	***0*.*29 (0*.*14–0*.*61)***	***0*.*002***
**Ethnicity**		
Han	Ref	
Other	1.50 (0.63–3.54)	0.348
**Annual household income (CNY)**		
< 30,000	Ref	
30,000–99,999	0.80 (0.39–1.65)	0.546
≥ 100,000	0.83 (0.38–1.82)	0.630
**Parental highest educational level**		
Middle school and below	Ref	
High school	0.93 (0.43–2.03)	0.855
College and above	0.94 (0.45–1.95)	0.865
**Acne vulgaris**		
Clear / mild	Ref	
Moderate / Severe	1.61 (0.78–3.36)	0.193
**Rosacea**		
Clear	Ref	
Rosacea	***3*.*22 (1*.*22–8*.*46)***	***0*.*019***

AOR: adjusted odds ratio.

### 3.3 Disease burden of AGA in college freshmen in China

According to Table 3, college AGA patients did not have higher burden of disease compared to those without alopecia. Utilities mapped by EQ-5D, symptoms of anxiety measured by GAD-2, symptoms of depression measured PHQ-2, and quality of sleep measured by PSQI were not significantly different between the groups (*P*>0.05).

**Table 3:** Quality of life in relation to androgenetic alopecia.

Measures	Present	Clear	*P*
Utilities mapping from EQ-5D	0.96±0.07	0.97±0.07	0.176
GAD-2	0.76±1.01	0.97±1.23	0.222
PHQ-2	0.80±1.08	0.90±1.26	0.580
PSQI	3.90±2.24	4.39±2.31	0.138

GAD: generalized anxiety disorder. PHQ: Patient Health Questionnaire. PSQI: Pittsburgh Sleep Quality Index.

## 4. Discussion

Our results revealed that the prevalence of AGA was 0.79% in male and 0.25% in female among college freshmen in China, which is much lower than a previous multiple-lateral research performed in six cities of China reporting that the prevalence of AGA was 2.8% in men and 1.3% in women aged 18–29 years [5]. This could be explained by the difference in age range between these two studies. The age distribution in our study (18.2±0.7) was significantly lower than previous study and the prevalence of AGA would be positively related to the increase of age. These discrepancy could also be attributed to the small proportion of adolescent among all AGA patients since another previous observation suggested that the fraction of adolescent has been found to be 3.5–6.5% among those hair-loss clinic visitors [9].

The sex ratio of male to female in our study was around 108:100. Our result revealed that male was significantly associated with higher prevalence of AGA (male 7.9/1000, female 2.5/1000, P<0.01). This gender composition is in line with previous studies showing the prevalence rate of AGA to be higher in males compared to females [5, 6, 8, 29–32]. Female was considered to have a lower risk of AGA and the extent of hair loss of women was generally less than in men [2]. Much evidence have proved that female may have a different pathophysiology from male [33, 34]. The findings of Sawaya and Price suggested that the level of 5a-reductase-anda-dronen receptors in frontal follicles was lower in women than men [35]. Female patients usually presented with normal androgens levels which suggested other multi-factorial androgen-independent mechanism [36–39]. Moreover, AGA was likely to affect postmenopausal women. A recent research from Starace, M et al. noted that estrogen could be the protective role on human hair growth [40]. Further work is required to explore the reason of the different prevalence between men and women.

According to previous studies, the association between AGA with BMI was discrepant. Some studies have shown that the BMI was higher in male AGA patients than normal control. Conversely, other studies have denied this relationship. Ding et al. reported the epidemiologic data regarding AGA in Chinese young adults which No association was found between high BMI (>25 kg/m2) and early-onset AGA [13]. In consistence, our results also suggested that there was no significant association between BMI and AGA. This might be because of that the participants in our study were all adolescents whose BMI were relatively confined. Moreover, our result might imply that in the absent of coronary heart disease, BMI alone might not be an independent risk factor of AGA.

Thorough dermatological examinations reveal that the most common comorbidities of AGA among adolescent were acne vulgaris and rosacea. The possible relationship between AGA and rosacea or acne was suggested by previous studies due to an underlying state of hyperandrogenism [14, 16]. To our surprise, our study suggested that only rosacea, other than acne vulgaris, were significantly associated with higher prevalence of AGA. Similar result was revealed by another study conducted among middle-aged AGA patients (men aged 40–69 years) which claimed no associations between AGA and acne vulgaris [41]. Our results also implied that rosacea was connected with AGA in a mechanism independent of hyperandrogenism. One possible explanation might be demodex mites which commensal lived on face [37, 42]. The mites often appear within pilosebaceous follicles. Qualitative studies demonstrated that demodex is a vital risk factor for rosacea and played an important role in the pathophysiology of rosacea [37, 43]. Similarly, the role of demodex mites in AGA has been suggested in some studies directly and indirectly [44]. An early research by Nioxin labs showed that 53 out of 54 patients presented with increased demodex mites in their alopecic scalps [45]. Nevertheless, a small scale study by Zari J draw different conclusions, denying the relation between AGA and Demodex mites [15]. Further work is needed to fully understand the possible relationship between AGA and rosacea.

Our results revealed that the HrQoL was almost unaffected among college students with AGA compared to those without AGA. This could be explained by the fact that most of college AGA patients were still in the early stage of disease when the hair loss was not apparent. Besides, most of the college AGA patients were male who were supposed to be less concerned about their appearance.

This current study has several limitations. Firstly, these questionnaires were based on self-report and the response rate was not satisfactory. Secondly, since the limited feasibility, we didn’t choose dermatology-specific quality-of-life measures such as the Dermatology Life Quality Index (DLQI) during the dermatological examination. EQ-5D-3L might not serve as a sensitive measure in detecting mild burden of many skin diseases owing to its strong ceiling effect. Despite these limitations, as far as we know, this is the first large population-based cross-sectional survey among college students. Moreover, the center effect was examined, and multilevel statistical model was used to estimate the associations. Since cluster sampling may introduce dependent variance structure, multilevel model can properly deal with the problem and provide unbiased estimation.

In conclusion, the onset of AGA in Chinese college students differs between genders and was significantly associated with rosacea.

## Supporting information

S1 Checklist STROBE statement—Checklist of items that should be included in reports of observational studies. (DOCX)
